# Global burden of potentially life-threatening maternal conditions: a systematic review and meta-analysis

**DOI:** 10.1186/s12884-023-06199-9

**Published:** 2024-01-02

**Authors:** Fitiwi Tinsae Baykemagn, Girmatsion Fisseha Abreha, Yibrah Berhe Zelelow, Abadi Kidanemariam Berhe, Alemayehu Bayray Kahsay

**Affiliations:** 1 Department of Nursing, College of Medicine and Health Sciences, Adigrat University, Tigray, Ethiopia; 2 School of Public Health, College of Health Sciences, Mekelle University, Tigray, Ethiopia; 3 Department of Obstetrics and Gynecology, School of Medicine, Mekelle University, Tigray, Ethiopia; 4 School of Public Health, College of Medicine and Health Sciences, Adigrat University, Tigray, Ethiopia; 5 Tigray Health Research Institute, Mekelle, Ethiopia

**Keywords:** Potential life-threatening, Severe maternal morbidity, Meta-analysis

## Abstract

**Background:**

Potentially life-threatening maternal conditions (PLTCs) is an important proxy indicator of maternal mortality and the quality of maternal health services. It is helpful to monitor the rates of severe maternal morbidity to evaluate the quality of maternal care, particularly in low- and lower-middle-income countries. This study aims to systematically identify and synthesize available evidence on PLTCs.

**Methods:**

We searched studies in English from 2009‒2023 in PubMed, the National Library of Medicine (NLM) Gateway, the POPLINE database, and the Science Direct website. The study team independently reviewed the illegibility criteria of the articles. Two reviewers independently appraised the included articles using the Joanna Briggs Instrument for observational studies. Disputes between the reviewers were resolved by consensus with a third reviewer. Meta-analysis was conducted in Stata version 16. The pooled proportion of PLTCs was calculated using the random effects model. The heterogeneity test was performed using the Cochrane Q test, and its level was determined using the I^2^ statistical result. Using Egger's test, the publication bias was assessed.

**Result:**

Thirty-two cross-sectional, five case–control, and seven cohort studies published from 2009 to 2023 were included in the meta-analysis. The highest proportion of PLTC was 17.55% (95% CI: 15.51, 19.79) in Ethiopia, and the lowest was 0.83% (95% CI: 0.73, 0.95) in Iraq. The pooled proportion of PLTC was 6.98% (95% CI: 5.98–7.98). In the subgroup analysis, the pooled prevalence varied based on country income level: in low-income 13.44% (95% CI: 11.88–15.00) I^2^ = 89.90%, low-middle income 7.42% (95% CI: 5.99–8.86) I^2^ = 99.71%, upper-middle income 6.35% (95% CI: 4.21–8.50) I^2^ = 99.92%, and high-income 2.67% (95% CI: 2.34–2.99) I^2^ = 99.57%. Similarly, it varied based on the diagnosis criteria; WHO diagnosis criteria used 7.77% (95% CI: 6.10–9.44) I^2^ = 99.96% at *P* = 0.00, while the Centers for Disease Controls (CDC) diagnosis criteria used 2.19% (95% CI: 1.89–2.50) I^2^ = 99.41% at *P* = 0.00.

**Conclusion:**

The pooled prevalence of PLTC is high globally, predominantly in low-income countries. The large disparity of potentially life-threatening conditions among different areas needs targeted intervention, particularly for women residing in low-income countries. The WHO diagnosis criteria minimize the underreporting of severe maternal morbidity.

**Trial registration:**

CRD42023409229.

**Supplementary Information:**

The online version contains supplementary material available at 10.1186/s12884-023-06199-9.

## Background

Potentially life-threatening conditions (PLTCs) refer to severe maternal morbidity found in women during pregnancy, childbirth, or in the puerperium including hypertensive disorders, hemorrhagic disorders, other systemic disorders, and indicators of severe management [1, 2], from which maternal near-miss conditions emerge [3].

Although there has been progress in decreasing maternal mortality worldwide, it is estimated that 295,000 maternal deaths still occur annually [4]. Almost 85% of those deaths occur in sub-Saharan African (SSA) countries [5]*.* More than 80% of all maternal deaths are caused by obstetric hemorrhage, hypertensive disorders of pregnancy, infection or sepsis, and unsafe abortions [6].

Maternal mortality is only the tip of the iceberg, setting above the poorly documented mass of maternal morbidities [7–9]. Severe maternal morbidities occur 23–30 times more frequently than maternal deaths [9, 10], and most cases share various characteristics with those women who do not survive [11–14]. Maternal mortality has been used to evaluate the quality of maternal healthcare services, but it is challenging to use this in situations when the absolute number of maternal deaths is infrequent or where conditions go unreported [2, 13]. As a result, there is increasing agreement on the use of monitoring the rate of potentially life-threatening conditions (PLTC) as an additional or alternative measure for assessing the effectiveness of maternal health care services [15, 16].

Severe maternal complications, including PLTC, are a major public health concern around the globe. Addressing all causes of maternal morbidity is one of the five key strategic objectives to achieve Sustainable Development Goal (SDG) 3.1, reducing the incidence of maternal mortality to < 70 per 100,000 live births by 2030 [17]. However, collected evidence is scarce on potentially life-threatening conditions. This knowledge gap was also noticed in another study [18].

For targeted maternal health, intervention requires an understanding of the magnitude of maternal morbidities. Despite the increasing number of studies on maternal near-miss [13, 19, 20], the proportion of PLTC remains relatively unclear.

To determine the prevalence of PLTCs in various nations around the world, numerous studies have been carried out. However, the majority of these studies found inconclusive findings. The prevalence of PLTCs in various studies conducted around the world ranged from 0.83% to 17.55% [21, 22]. Additionally, the majority of the published research used small sample sizes and only one study site. There is no worldwide study about the prevalence of PLTC. The results of this study will be important in developing better health policies for preventing PLTCs and better prevention strategies that can target the high prevalence of maternal conditions. Therefore, this study aimed to evaluate the pooled prevalence of potentially life-threatening maternal conditions worldwide.

## Methods

### Protocol and registration

We developed the research protocol based on the Preferred Reporting Items for Systematic Reviews and Meta-Analysis Protocols (PRISMA-P) 2020 checklist [23]. For details, see Additional File 1. The study selection process followed three phases, as shown in the PRISMA-2020 flow diagram [23]. The protocol of this study was registered in the International Prospective Register of Systematic Reviews (PROSPERO) (ID: CRD42023409229).

### Eligibility criteria

We included studies that reported the prevalence of potentially life-threatening conditions or data that could be used to calculate them. All studies published from January 1, 2009, up to June 2023 were included. The year 2009 was considered since the World Health Organization (WHO) maternal working groups developed the standard identification criteria for PLTC [2]. We excluded studies with no data on the prevalence of potentially life-threatening conditions, articles published in a language other than English, articles published before 2009, qualitative studies, systematic reviews, and case report studies.

The outcome variable of this study is the pooled prevalence of PLTC, which is defined as a maternal condition that fulfills at least one of the WHO/CDC. The WHO identification criteria include (i) hemorrhagic disorders; (ii) hypertensive disorders; (iii) other system disorders including sepsis; and (iv) severe management indicators during pregnancy, childbirth, or the postnatal period [24]. The CDC-indexed identification criteria for SMM do not include prolonged postpartum hospital stay and admission of any blood product as compared to WHO identification criteria [25]. All women during pregnancy, childbirth, or 42 days after pregnancy termination were the study population of this systematic review and meta-analysis.

### Information sources

International databases such as PubMed, the National Library of Medicine (NLM) Gateway, POPLINE, Google Scholar, and the Science Direct website were searched. Our initial search was conducted in November 2022 by the corresponding author (FT). A last search was conducted in June 2023 to ascertain any further studies published since our initial search. Backward and forward citation searching was used in Google Scholar.

### Search strategy

We developed Medical Subject Heading (MeSH) and ‘text word’ using different Boolean operators OR, AND, and NOT. In detail, the keywords used in the search are attached in the annex (see Additional File 2). In addition, we used the citing reference search (backward and forward) mechanism. The search was limited to the English language and studied after January 2009.

### Study selection

The citations identified in the search were exported into EndNote bibliography management software; then, duplicate studies were removed. The remaining citations were screened by title or abstract, and ineligible articles were excluded. The full-text articles were included if they reported the prevalence of PLTC or if they reported the total sample size and number of PLTC cases. Two authors (FT and GF) independently screened the selected articles using prespecified inclusion criteria. During the selection process, disagreements between two reviewers were resolved through discussion or input from other reviewers. The selection process was presented based on the PRISMA flow diagram 2020 [26].

### Data collection process and data items

Two independent reviewers (FT and GF) extracted the data. We contacted the first authors via email and asked them to provide the missing outcome data. During the data collection process, disputes between two reviewers were resolved through discussion or input from another reviewer (YB). Data on the outcome and other variables were extracted using a predefined Excel spreadsheet, such as first author, publication year, location of study, study population, study extent, diagnosis criteria, study design, sample size, study setting, sampling method, data collection method, data analysis, the prevalence of PLTC, *P* value, and 95% CI (see Additional File 3).

The level of agreement between the independent data extractors (FT and GF) was calculated using kappa statistics to show the difference between the expected and observed agreement. The Kappa value was 96%, suggesting almost perfect agreement, according to Viera et al. [27].

### Quality assessment

We used the Joanna Briggs Institute Meta-Analysis of Statistics Assessment and Review Instrument (JBI-MAStARI) to assess the quality of the included studies based on their type of study design [28]. This quality assessment instrument in each study design has 11 criteria in a cohort, 10 criteria in a case–control study, and 8 criteria in a cross-sectional study. For each criterion, if "yes," we gave a score of one; otherwise, we gave a zero score, which means an answer of "no, "not applicable, or "not clear''. Two reviewers independently evaluated the risk of bias for each article. Disagreements between reviewers were resolved through discussion and input from a third reviewer. Finally, the risk of bias was considered low when ≥ 70% of the answers were ‘yes’, moderate when 50–69% were ‘yes’, and high when < 49% were ‘yes’[29].

### Data analysis

The characteristics of the included studies were synthesized in the text and summarized in tables. Stata version 16.0 software was used to analyze the data. Meta-analysis was performed to estimate the pooled prevalence of PLTC with a 95% confidence interval. The prevalence of PLTC was calculated by dividing the number of women who had PLTC by the total number of women who have been included in the study multiplied by 100. Thus, the outcome measure was computed with ‘metaprop’, a stata command for meta-analysis of prevalence. We generated forest plots to show the individual studies as well as the pooled prevalence of PLTC with 95% CI.

### Heterogeneity test

The heterogeneity test was assessed using Cochrane’s Q test and quantified with I^2^ statistics. A *P* value less than 0.05 was considered the cutoff point for heterogeneity. The level of heterogeneity was determined as low if < 25%, moderate when 25–75%, and high when > 75% [30]. We used the random effect model for pooling PLTC because studies anticipated heterogeneity. A meta-regression analysis was carried out to investigate the sources of heterogeneity based on the study design, diagnostic criteria, country income level, publication year, study extent, and sample size.

### Assessment of publication bias

A funnel plot was used to evaluate publication bias, which is the tendency to publish research that has positive results or that has statistically significant findings [31]. An asymmetrical graph was considered to suggest a publishing bias, and vice versa, based on the shape of the graph [32]. We conducted a counter-enhanced funnel plot to differentiate between publication bias and another cause of funnel plot asymmetry, such as actual heterogeneity between large and small studies (the small study effect) and variations in baseline characteristics in the included studies [33]. Moreover, to test for publication bias, we used Egger's weighted regression; a *p*-value less than 0.05 was considered to suggest the presence of statistically significant publication bias [32].

### Subgroup analysis

We performed subgroup analysis based on various study characteristics, including sample size, diagnostic criteria used (WHO or CDC), the five-year interval of publication (2013–2017 vs. 2018–2022), study country income based on the World Bank (low, low-middle, upper-middle, and high income), and the sample size.

### Sensitivity analysis

To determine how much an alteration in the study methodology affected the meta-analysis’s results, we conducted a sensitivity analysis. This helped in evaluating the one study sample size on the overall results. In specific, the leave-one-out analysis was used, in which one primary study was excluded at a time [34, 35]. Then we compared the new pooled PLTC with the original PLTC. When the new pooled PLTC was found to lie outside of the 95% confidence interval of the original pooled PLTC value, we concluded that the excluded study had a significant effect on the meta-analysis study and should be excluded from the last analysis. However, we didn't find any studies that lay outside of the initial 95% CI.

## Results

### Study selection

**Fig. 1:**
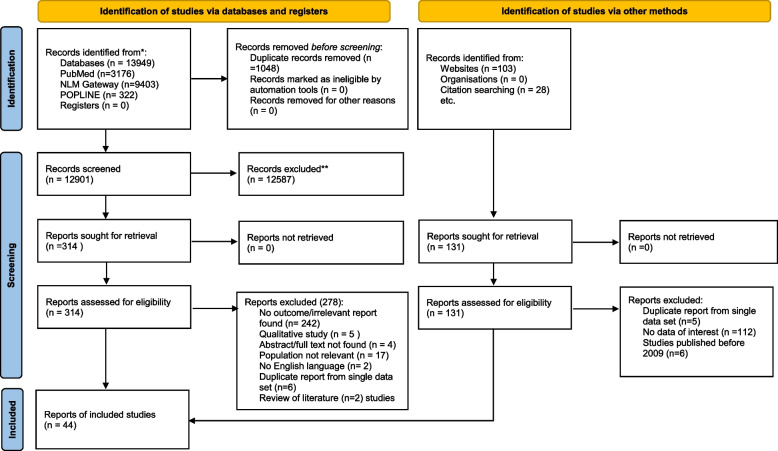
PRISMA flow chart showing identification and selection of studies

A total of 13,949 citations were identified through the electronic database search using the aforementioned search terms. After removing duplicate citations using EndNote software, 12901 studies remained. Out of these, 12587 were excluded by titles or abstracts, leaving 314 for the full-text evaluation. Subsequently, 278 articles were excluded: irrelevant or didn’t report the main outcome (*n* = 242), populations not relevant or high-risk women (*n* = 17), qualitative studies (*n* = 5), conference abstracts (*n* = 4), non-English language (*n* = 2), review of literature (*n* = 2), and duplicated reports from a single data set (*n* = 6). Additionally, 131 studies were identified using the website and citation searches; after excluding irrelevant studies, 8 reports were included. The process of inclusion and exclusion is detailed in the PRISMA flow diagram 2020 (see Fig. 1).

In total, 44 studies [3, 22, 24, 36–76] provide data on the prevalence of PLTC. The studies were conducted in 17 different countries; in addition, four studies had multiple country sites [46, 67, 71, 72]. The countries with the largest number of included studies comprised Brazil (*n* = 9), India (*n* = 7), Ethiopia (*n* = 5), the United States (*n* = 3), Malaysia (*n* = 2), and South Korea (*n* = 2). The remaining studies are one each from 11 countries. All the included studies were observational 32 cross-sectional [3, 22, 24, 36–58, 67, 72–76], 5 case–control [66, 68–71], and 7 cohort study designs [59–65]. All the reviewed studies were published between 2012 and 2022. The extent of the included study area was: 19 in a single site, 13 in two or more sites, 6 nationally, and 6 in network-type (multicountry) studies. Eighty-six percent of the included studies used the World Health Organization’s (WHO) diagnosis criteria for PLTC, and only 13.64% of studies used the Centers for Disease Control’s (CDC) criteria. In this meta-analysis, a total of 4158663 study participants were included. The Minimum (0.83%) and maximum (17.55%) prevalences of PLTC were reported in Iraq [53] and Ethiopia [37], respectively. For more detailed information on each article, (see Table 1).

**Table 1:** Characteristics of included studies (*N* = 44)

Author, publication year	Study Country	Study Extent	Diagnosis criteria	Study Desing	Sample size	PLTC(%)
**Rajbanshi et al. 2021** [36]	Nepal	Single site	WHO	CS	346	6.60
**Murki et al. 2017** [57]	India	Single site	WHO	CS	1127	11.09
**Tenaw et al. 2021** [22]	Ethiopia	2 + sites	WHO	CS	1214	17.55
**Woldeyes et al. 2018** [74]	Ethiopia	Single site	WHO	CS	2737	13.30
**Tunçalp et al. 2014** [16]	Gahna	Single site	WHO	CS	3438	15.00
**Tunçalp et al. 2013** [49]	Gahna	Single site	WHO	CS	3438	15.01
**Tallapureddy et al. 2017** [54]	India	Single site	WHO	CS	3900	4.72
**Teka et al. 2022** [47]	Ethiopia	Single site	WHO	CS	5116	13.5
**Herklots et al. 2017** [75]	Tanzania	Single site	WHO	CS	5551	10.3
**Hitti et al. 2018** [76]	United States	Single site	CDC/ICD 9–10	CS	7025	4.00
**Roopa et al. 2013** [48]	India	Single site	WHO	CS	7390	10.2
**Francisco et al. 2018** [55]	Brazil	Single site	WHO	CS	8077	2.70
**Menezes et al. 2015** [58]	Brazil	2 + sites	WHO	CS	20435	5.85
**Norhayati et al. 2016** [3, 39]	Malaysia	2 + sites	WHO	CS	21579	1.83
**Norhayati et al. 2016** [3, 39]	Malaysia	2 + sites	WHO	CS	23422	1.69
**Jabir et al. 2013** [21]	Iraq	2 + sites	WHO	CS	25472	0.83
**Chb et al. 2015** [56]	South Africa	2 + sites	WHO	CS	26614	4.21
**Aleman et al. 2022** [72]	LACs	Network	WHO	CS	33901	8.00
**Tan et al. 2015** [53]	China	2 + sites	WHO	CS	33993	4.34
**Owolabi et al. 2020** [73]	Kenya	National	WHO	CS	36162	5.50
**Moreira et al. 2017** [67]	Brazil	National	WHO	CS	36724	5.60
**Balachandran et al. 2022** [51]	India	Single site	WHO	CS	37590	4.88
**Ghazivakili et al. 2016** [45]	Iran	2 + sites	WHO	CS	38715	1.08
**Maity et al. 2022** [50]	India	Single site	WHO	CS	39310	4.50
**Ba, Anna, et al. 2021 **[52]	United States	2 + sites	CDC/ICD 9–10	CS	48608	1.50
**Oliveira et al. 2014** [44]	Brazil	Network	WHO	CS	82144	10.52
**Santana et al. 2017** [37]	Brazil	Network	WHO	CS	82388	10.01
**Zanardi et al. 2020** [77]	Brazil	Network	WHO	CS	82388	11.60
**Santana et al. 2018** [24]	29 WHOMC	Network	WHO	CS	287077	6.20
**Reid et al. 2018** [38]	United States	National	CDC/ICD 9–10	CS	364113	1.97
**Serruya et al.** 2017 [46]	LACC	2 + sites	WHO	CS	712081	15.50
**Dzakpasu et al.** 2020 [40]	Canada	National	CDC/ICD 9–10	CS	1418545	1.61
**Pacheco et al. 2014** [59]	Brazil	Single site	WHO	Cohort	2291	17.50
**Beyene et al. 2022** [63]	Ethiopia	2 + sites	WHO	Cohort	3006	10.40
**Magar et al. 2020** [62]	India	Single site	WHO	Cohort	4351	6.91
**Tura A. et al. 2018** [64]	Ethiopia	2 + sites	WHO	Cohort	7929	13.30
**Crom et al. 2016** [60]	Itali	Single site	WHO	Cohort	23453	6.30
**Nam et al. 2019** [61]	South Korea	National	CDC/ICD 9–10	Cohort	90072	2.31
**Nam et al. 2022** [65]	South Korea	National	CDC/ICD 9–10	Cohort	280612	2.30
**Fauconnier et al. 2020** [71]	FBS	Network	WHO	CC	3825	3.40
**Madeiro et al. 2015** [68]	Brazil	Single site	WHO	CC	5841	5.87
**Paes et al. 2014** [66]	Brazil	Single site	WHO	CC	16243	7.30
**Chhabra et al. 2019** [70]	India	Single site	WHO	CC	38111	1.80
**Raineau et al. 2022** [69]	France	2 + sites	WHO	CC	182309	1.40

*WHOMC* WHO multicenter countries, *CS* Cross-Sectional, *CC* Case–Control, *LACs* Latin American Countries, *LACC* Latin America and Caribbean countries, *FBS* France, Belgium, and Switzerland

### Risk of bias assessment

Overall, 44 studies underwent quality assessment and all had low risks of bias. The quality appraisal scores mean (± SD) of the included studies was 6.69 (± 0.97) for cross-sectional, 9.40 (± 0.55) for case–control, and 9.86 (± 1.07) for cohort study design. All articles were explored in the systematic review and meta-analysis. For the detailed score of each study (see Additional File 4).

### Prevalence of potentially life-threatening maternal conditions

**Fig. 2:**
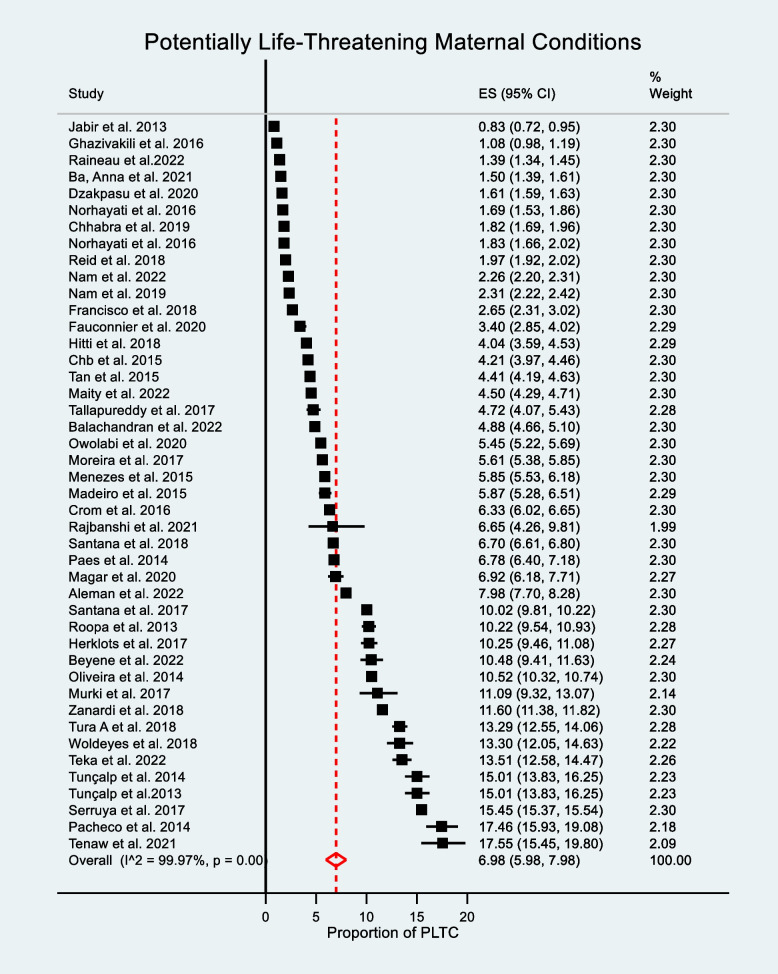
Forest plot showing the pooled prevalence of potentially life-threatening conditions

The pooled prevalence of PLTC was 6.98% (95% CI: 5.98–7.98). A random-effects model was used due to the presence of significant heterogeneity in the included studies (I^2^ = 99.97%, *P* = 0.00). The prevalence ranged from 0.83% (95% CI: 0.73–0.95) in Iraq to 17.55% (95% CI: 15.51–19.79) in Ethiopia, as shown in Fig. 2.

### Subgroup analysis

We performed subgroup analysis to identify the sources of heterogeneity using different characteristics: diagnosis criteria (WHO vs. CDC/ICD9-10), country income level (low, lower-middle, upper-middle, and high-income country), publication year (2013–2017 vs. 2018–2022), and sample size (> 20000 vs. ≤ 20000) (see Table 2).

**Table 2:** Pooled prevalence,95% CI, and heterogeneity level of PLTC by subgroup analysis

Subgroup	Number of studies	Sample size	Number of PLTC	Prevalence of PLTC (95% CI)	I^2^,%	*P*-value	Weight %
Diagnosis criteria							
**WHO**	38	1949688	183844	6.88 (5.09–8.91)	99.96	0.00	86.18
**CDC/ICD9-10**	6	2208975	39399	2.19 (1.89–2.50)	99.41	0.00	13.82
Country income level							
**Low income**	5	20002	2637	13.44 (11.88–15.00)	89.90	0.00	11.08
**Low-middle**	12	215991	9160	7.42 ( 5.99–8.86)	99.71	0.00	29.21
**Upper-middle**	14	467611	35388	6.35 (4.21–8.50)	99.92	0.00	32.09
**High income**	9	2418562	43553	2.67 (2.34–2.99)	99.57	0.00	20.71
Publication Year							
**2013–2017**	22	1225848	148495	8.57 (5.79–11.34)	99.97	0.00	49.91
**2018–2022**	22	2932815	74748	5.31 (4.71–5.91)	99.89	0.00	50.09
Study sample size							
**≤ 20000**	19	92845	8099	9.86 (8.00–11.73)	99.22	0.00	42.43
**> 20000**	25	4065818	215144	4.27 (3.56–6.18)	99.98	0.00	57.57

*CDC* Center for Disease Control, *WHO* World Health Organization, *ICD9* International Classification of Disease Code9

**Fig. 3:**
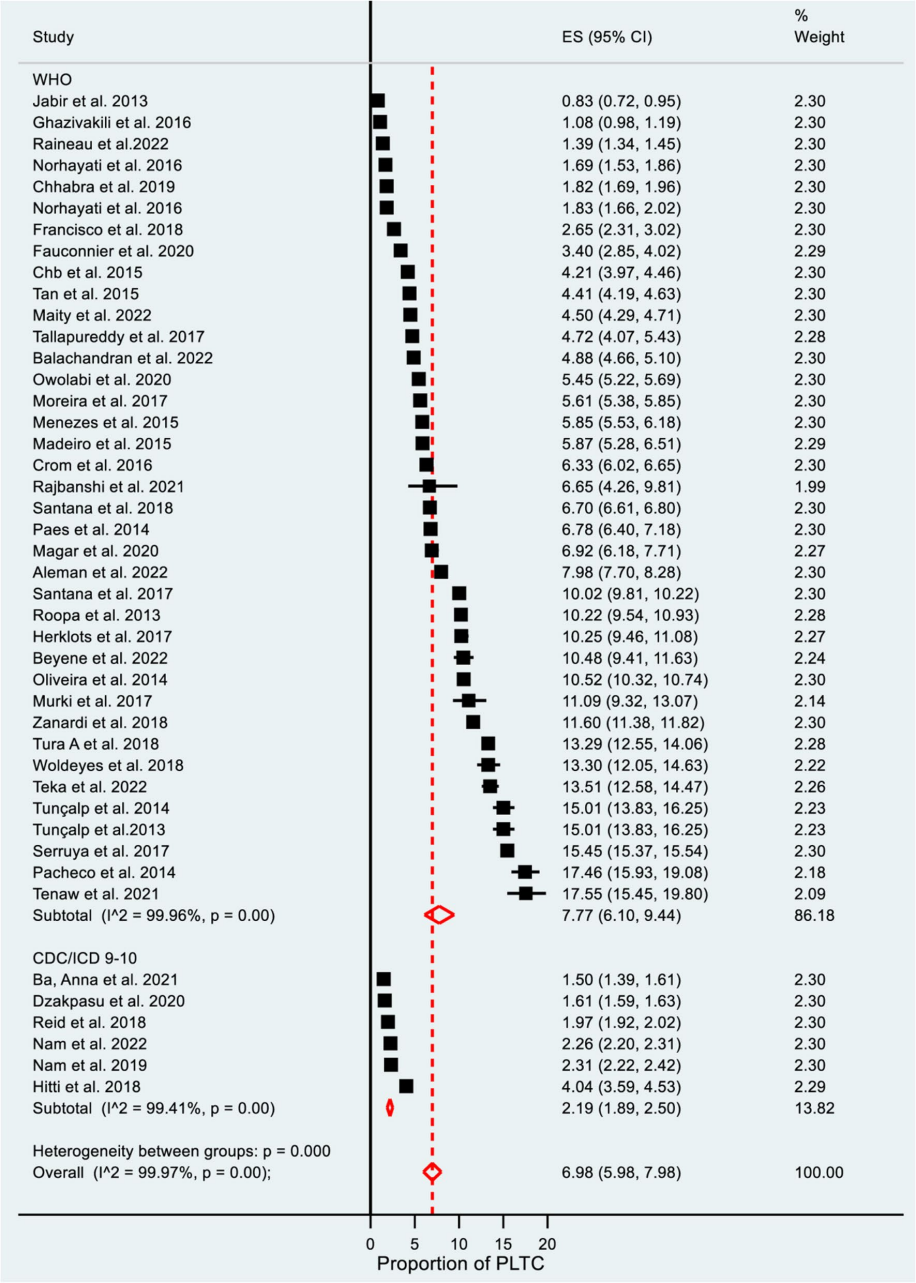
Subgroup analysis for the prevalence of potentially life-threatening conditions by diagnostic criteria

**Fig. 4:**
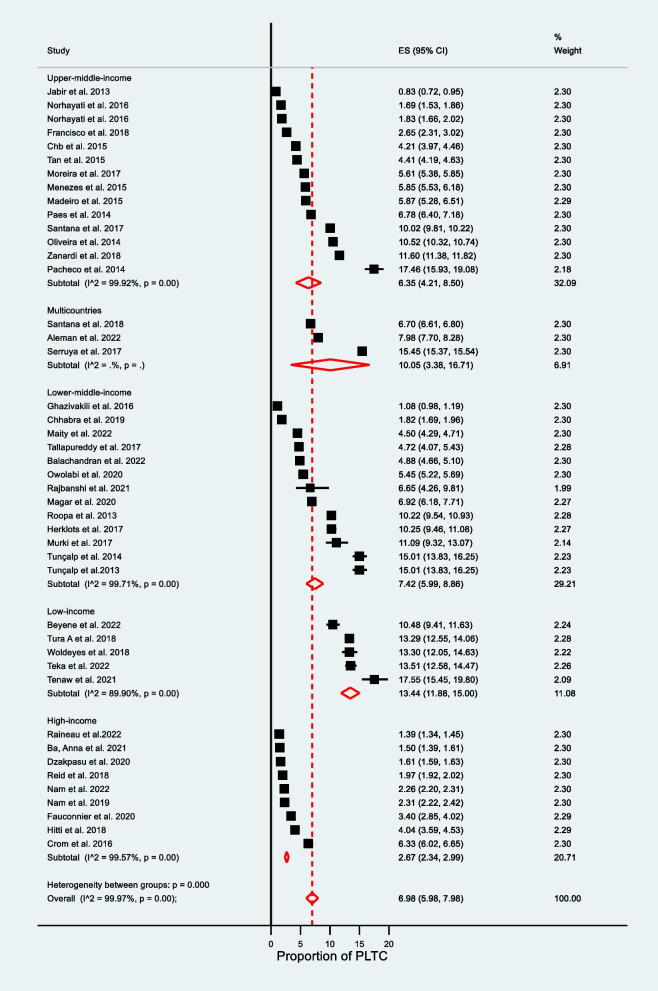
Subgroup analysis for the prevalence of potentially life-threatening conditions by economic level of countries

**Fig. 5:**
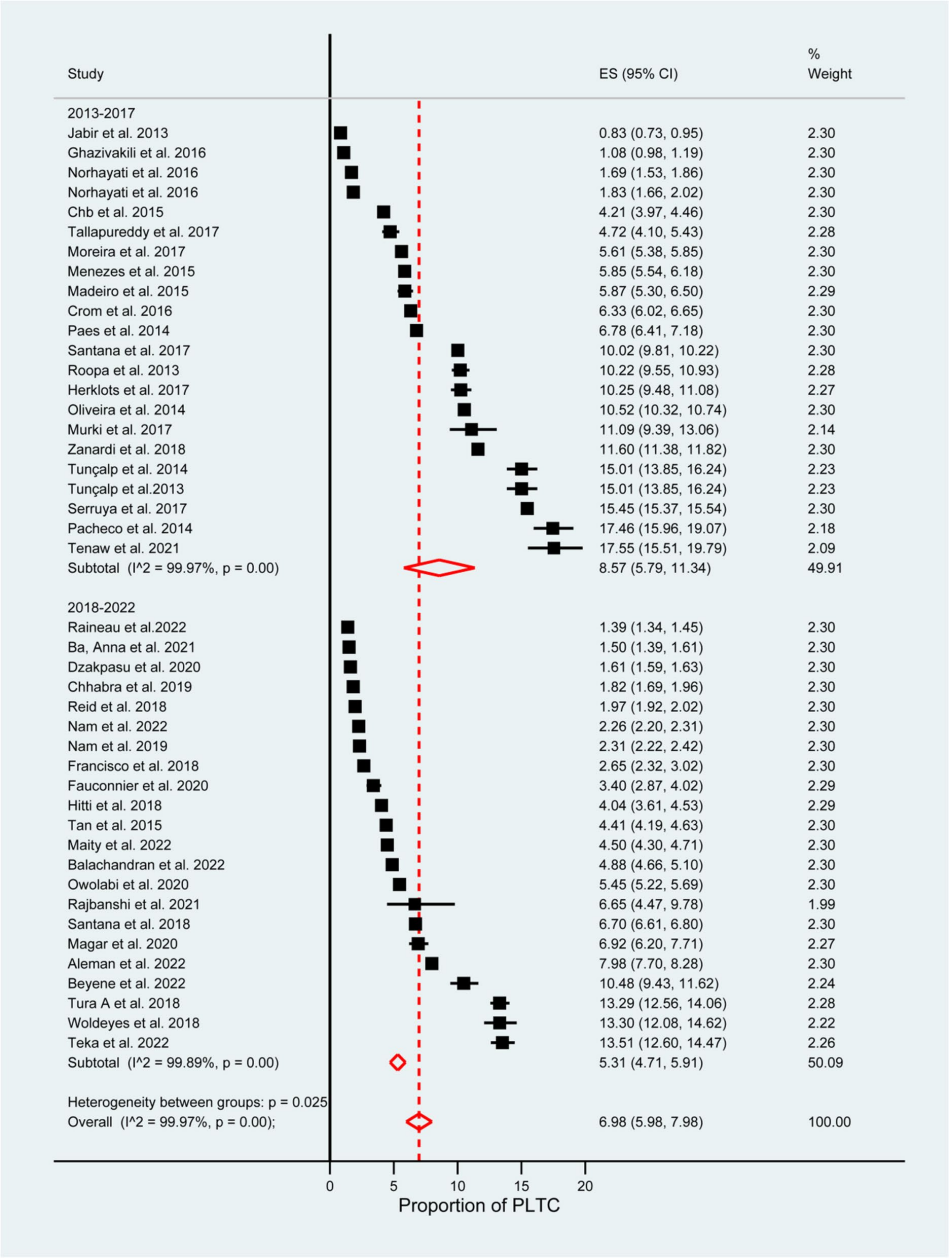
Subgroup analysis for the prevalence of potentially life-threatening conditions by year of publication

Accordingly, the pooled prevalence for WHO diagnostic criteria used was higher at 7.77% (95% CI: 6.10–9.44), at I^2^ = 99.96%, and *P* = 0.00 as compared to CDC diagnosis criteria used at 2.19% (95% CI: 1.89–2.50), at I^2^ = 99.41%, and *P* = 0.00 (see Fig. 3). The CDC-indexed diagnosis criteria are fewer in number as compared to WHO diagnosis criteria (do not include blood transfusion and prolonged postpartum hospital stay. The WHO minimizes the underreporting of PLTCs. The pooled prevalence varied based on country income level: in low-income countries, 13.44% (95% CI: 11.88–15.00), at I^2^ = 89.90%; in low-middle income countries, 7.42% (95% CI: 5.99–8.86) I^2^ = 99.71%; in upper-middle-income countries, 6.35% (95% CI: 4.21–8.50) at I^2^ = 99.92%; and in high-income countries, 2.67% (95% CI: 2.34–2.99) at I^2^ = 99.57% (see Fig. 4). Publication year 2013–2017 was significantly higher 8.57% (95% CI: 5.79–11.34) I^2^ = 99.97% as compared with studies published 2017–2022 [5.31% (95% CI:4.71–5.91) I^2^ = 99.89% at *P* = 0.00] (see Fig. 5).

**Fig. 6:**
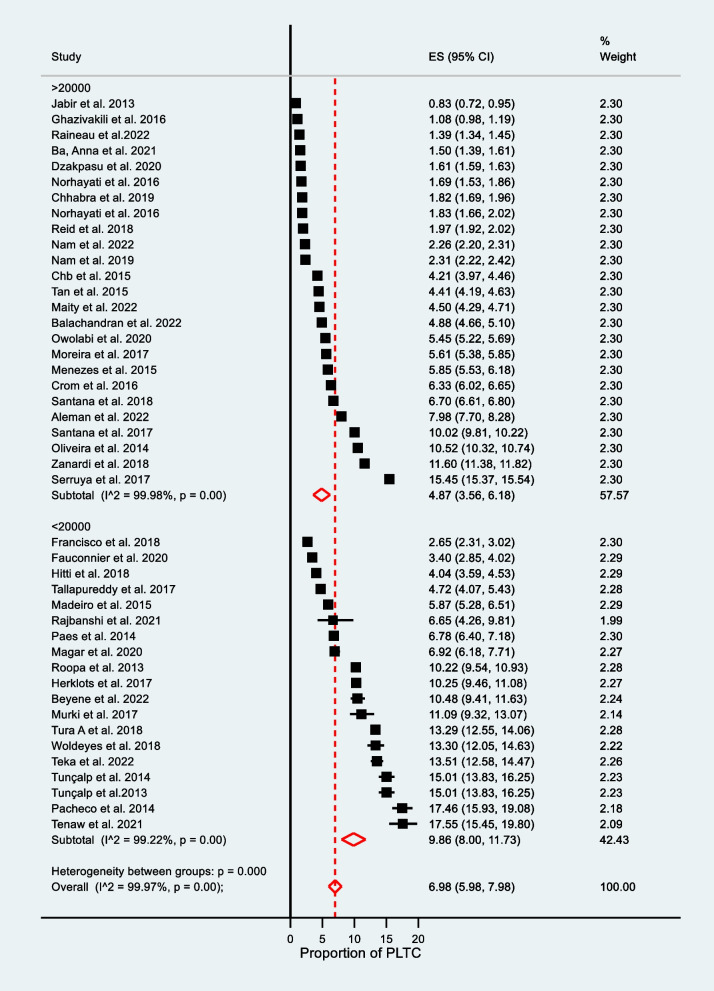
Subgroup analysis for the prevalence of potentially life-threatening conditions by study sample size

Similarly, the pooled prevalence based on sample size (≤ 20000) was 9.86% (95% CI: 8.00–11.73), I^2^ = 99.18% in comparison with study sample size (> 20000) of 4.87% (95% CI: 3.56–6.18), I^2^ = 99.98% (see Fig. 6).

### Publication bias and sensitivity analysis

The funnel plot had asymmetry, which suggested a lack of precision in prevalence estimates, possible publication bias, and high heterogeneity (Additional File 5). In addition, the Egger test for small study effects resulted in a significant result (*P* < 0.001).

Sensitivity analysis was carried out by sequentially removing studies (the leave-one-out) to evaluate the effect of sample size on the result of the meta-analysis. We found that no single study lay outside of the 95% CI of the original pooled PLTC; we concluded that the excluded study had no significant effect. (see Additional File 6).

### Time trend analysis

**Fig. 7:**
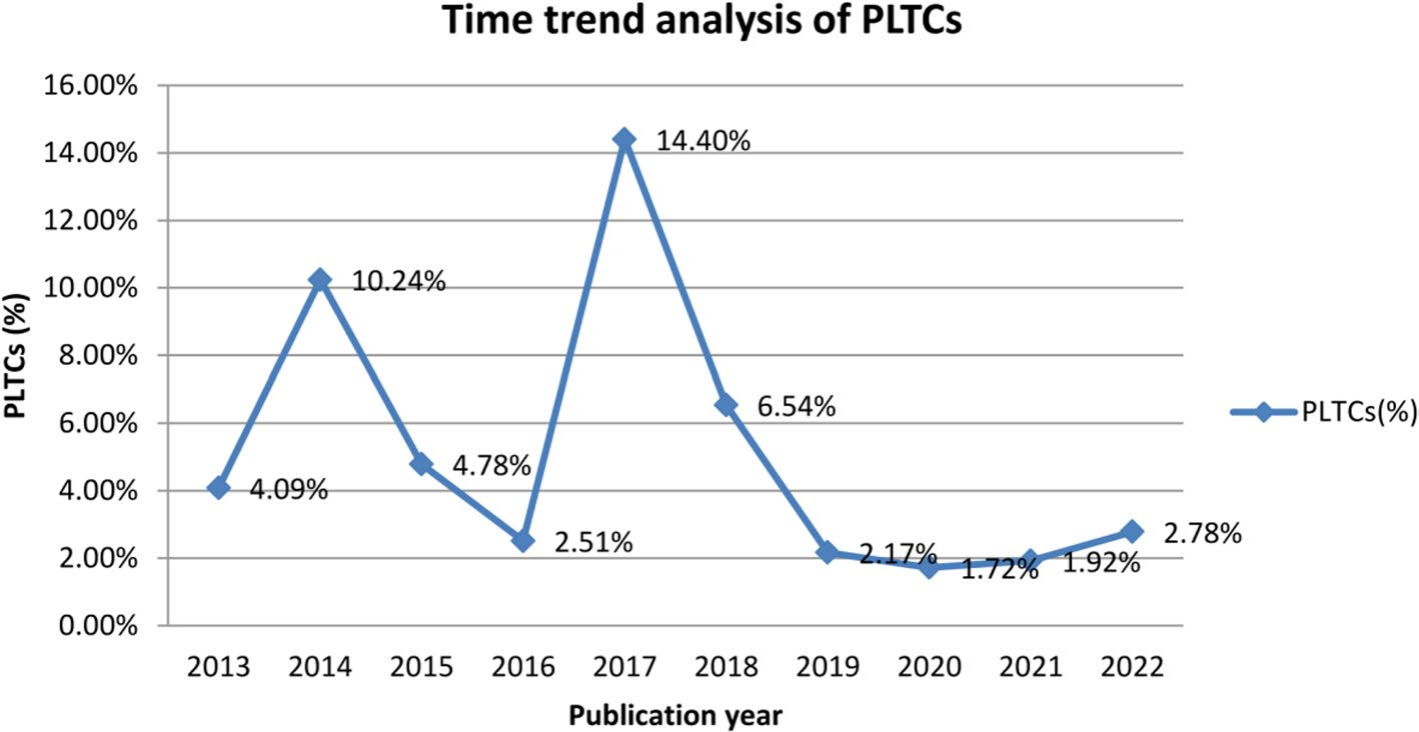
Time trend prevalence of potentially life-threatening conditions from 2013 to 2022

The time trend analysis indicated the pooled prevalence of PLTC for every year, which is calculated by adding the number of PLTC cases from each study in the same year divided by the total sample size of the studies in that year. In the time trend analysis, the minimum (two studies) and maximum (seven studies) were included in the years 2017 and 2022 respectively The trends of PLTCs increased between 2013 and 2014, decreased between 2014 and 2016, increased in 2017, decreased between 2018 and 2020, and increased between 2021 and 2022, The graph showed a slight decrease in PLTCs over the past 10 years. Nevertheless, we found no statistically significant variation in the time trend analysis (*P* = 0.28) over the last 10 years. For more detail (see Fig. 7).

### Meta-regression

A meta-regression analysis was performed to determine the potential sources of heterogeneity using diagnosis criteria, the economic level of the study country, the study publication year, and sample size. The univariate regression analysis showed PLTC increased by WHO diagnosis criteria, with statistically significant differences. The univariate meta-regression model revealed that the WHO diagnosis criteria explained more than 20% of between-study heterogeneity. Other characteristics of the primary study that explained the study’s heterogeneity were the country's economic level (15%) and the sample size (18%) (see Table 3).

**Table 3:** Univariate and multivariable meta-regression analysis involving different study characteristics

Study characteristics	Univariate meta-regression analysis			
	**coefficients**	**95%CI**	***P*-value**	**Explained heterogeneity R**^**2**^** (%)**
**Diagnosis criteria (WHO VS. CDC/ICD)**	5.52	1.59 to 9.44	0.01	20.79
**Country economic level (ref-High income)**				15.82
** low income**	10.85	6.57 to15.14	0.00	
** Low-middle**	4.75	1.42 to 8.08	0.01	
** Upper middle**	3.66	0.38- 6.94	0.03	
**Sample size (ref: > 20000)**	-5.08	-7.60 to -2.55	0.00	17.92
**Multivariable regression analysis**				14.51
** Diagnosis criteria (CDC/ICD VS. WHO)**	-0.84	-6.14 to 4.46	0.76	
**Country economic level (ref-High income)**				
** low income**	4.41	0.30 to 8.52	0.04	
** Low-middle**	-0.19	-3.03 to 2.65	0.89	
** Sample size (ref: ≤ 20000)**	3.46	0.76 to 6.16	0.01	

R^2^: regression goodness of-fit index: % of explained (by covariate) heterogeneity on total heterogeneity

The multivariable regression model included all the variables that were significantly related to PLTC prevalence, diagnostic criteria, country income level, and study sample size. However, in the multivariate regression model, none of the covariates tested for sources of heterogeneity were significant. Therefore the heterogeneity could be explained by other variables not included in this meta-analysis study (see Table 3).

## Discussion

This systematic review suggests that the global pooled prevalence of PLTCs is 6.98%. The prevalence of PLTC in low- and low-middle-income countries is the highest. We reviewed different studies that reported a wide range of PLTCs, from 0.83% to 17.55%. In this review, WHO identification criteria produced higher rates than the CDC criteria.

The systematic review highlighted the characteristics of the study, such as study design, sample size, sampling method, data collection methods, study setting, quality, and study distributions. The review included 44 different studies from different countries. One critical gap identified in this systematic review was the low number of studies [5] in low-income countries.

We compiled the proportion of PLTC from a vast sample size (4,158,663). Our findings suggested that the pooled prevalence of PLTC was 6.89% (95% CI: 5.98–7.98). The prevalence is found to be almost parallel with WHO reports of 7.0% [78]. The proportion of the current study is higher than that of a systematic review and meta-analysis conducted in Iran: 2.5/1000 live births [20]. The difference in prevalence is because this study has an international scope, but that study focused on Iran. Other differences may be associated with variables such as the diagnostic criteria used and the preexisting conditions of the women participating in the studies.

It was seen in this meta-analysis that PLTC prevalence varied according to countries' income levels, diagnosis criteria, publication year, and sample size. This finding provides a more comprehensive picture of the burden of PLTC, which can be used to target improvements in maternal health services. Although data are scarce in low-income countries, the proportion of PLTCs is associated with economic level. It was highest in low-income countries at 13.43% (11.89–15.04), followed by low-middle income at 7.42 (5.99–8.86), and lowest in high-income countries at 2.56% (2.15–3.01), which is consistent with prior systematic reviews carried out in a particular region [13, 19, 79]. This high prevalence in low-income countries may be associated with the low quality and coverage of maternal care [80]. This is supported by a systematic review conducted in developing countries and a WHO report, which found that women with a high-income level have better access to mass media, which increases the utilization of maternal health services [81, 82].

In this study, the proportion of PLTCs was higher in the WHO identification criteria than in the CDC/ICD9 indexed criteria. Souza et al. [78] reported similar results. WHO diagnosis criteria are used to minimize the underreporting of cases in clinical settings [2, 83]. It is recommended as an identification criterion, especially in low-resource settings [84]. In light of these results, it can be said that PLTC prevalence may vary according to the diagnostic criteria used. Another reason may be the entity of diagnostic criteria in WHO is more than the CDC identification criteria. the WHO criteria include any type of blood transfusion and prolonged postpartum length of stay in the hospital, but those are not included in CDC criteria [2, 25, 85].

The PLTC prevalence was lower in recently published studies (from 2018–2022). Similarly, Oladapo et al. [86] reported that the trend of severe maternal morbidity has decreased in recent years. The reason may be associated with improved coverage and quality of maternal care [87]. Hirai et al. [88] reported that the prevalence of severe maternal morbidity was higher in recent years. The difference may be associated with increased preexisting medical conditions and obesity [89].

The prevalence of PLTC in this study varied based on sample size, and a larger sample size had a lower prevalence than lower sample size studies. This finding is in line with another study conducted by DeSilva M et al. [19]. This may be because of representativeness or generalizability differences.

Important covariates of PLTC prevalence heterogeneity sources tested in the univariate meta-regression were diagnostic criteria, gross economic level of the study country, and sample size of the study. The contribution of these covariates was not confirmed by the results of multivariate meta-regression models.

This study has some limitations that should be noted. First, there was publication bias because we only included English studies. Second, the majority of the research in this review had a retrospective cross-sectional study design (secondary data), which might lack quality data. Third, the included studies had high heterogeneity. Fourth, does not include grey literature. Despite these limitations, the study has some strengths. First, we made a special effort to reach out to the authors for further information and clarification. Second, it is comprehensive in its scope. Third, it has additional analyses such as subgroup analysis, sensitivity analysis, and meta-regression.

## Conclusion and recommendations

There is a high prevalence of potentially life-threatening maternal conditions globally, and predominantly low-income countries are disproportionately affected. We have highlighted the utility and strength of severe maternal morbidity as a tool to measure the quality of maternal health care, especially in LMICs where maternal mortality data are deficient or lacking. Using the WHO diagnostic identification criteria, there was a high probability of PLTC detection.

The findings are used to inform maternal health policy and direct resources to improve maternal outcomes. This study provides an opportunity to implement targeted interventions that could have a major clinical impact. Safe and effective preventive and therapeutic maternal health interventions have to be equally accessible to all women. To minimize the underreporting of PLTC, the WHO identification criteria should be used.

### Supplementary Information

**Additional file 1.**

**Additional file 2.**

**Additional file 3.**

**Additional file 4.**

**Additional file 5.**

**Additional file 6.**

## Data Availability

All data generated or analysed during this study are included in this published article [and its supplementary information files].

## Abbreviations

CDC: Centers for Disease Controls
CI: Confidence Interval
ICD9-10: International classifications of disease code nine or ten
JBI-MAStARI: Joanna Briggs Institute Meta-Analysis of Statistics and Review Instrument
LMICs: Low and Middle-Income Countries
OR: Odds Ratio; SDG: Sustainable Development Goal
PLTC: Potentially life-threatening maternal conditions
PRISMA-P: Preferred Reporting Items for Systematic Reviews and Meta-Analysis
SDG: Sustainable development goal
SSA: Sub-Saharan African countries
WHO: World Health Organization
