# Supplementary material for: Global burden of potentially life-threatening maternal conditions: a systematic review and meta-analysis
Source: BMC Pregnancy Childbirth. 2024 Jan 2;24:11. doi: 10.1186/s12884-023-06199-9 (PMC10759711; doi:10.1186/s12884-023-06199-9)
Supplement: Supplementary file 2 — Additional file 2. [file 12884_2023_6199_MOESM2_ESM.pdf]

S2 File: Searching Keyword and MeSH terms for potentially life-threatening conditions

1. Sample search strategy from PubMed database

((((((((((((((Potentially[Text Word]) OR Severe[Text Word]) AND (life-threatening[Text Word])) OR (Potentially life-threatening conditions[Text Word])) OR (Potentially lifethreatening maternal conditions[Text Word])) OR (PLTC[Text Word])) OR (severe maternal morbidity[Text Word])) OR (severe obstetric complication[Text Word])) OR (Maternal morbidity[Text Word])) OR (maternal complications[Text Word])) OR (emergency obstetrics[Text Word])) OR (SMM[Text Word])) OR (Intensive care unit admission[Text Word])) OR (Acute maternal complication[Text Word])) OR (maternal conditions[Text Word])) AND (((((prevalence[MeSH Terms]) OR (Magnitude[Text Word])) OR (incidence[MeSH Terms])) OR (Proportion[Text Word])) AND (2009:2023[pdat]))

Number of articles searched = 3176

2. Sample search strategy from the National Library of Medicine (NLM) Gateway

(Potentially[All Fields] AND ("life"[MeSH Terms] OR "life"[All Fields]) AND threatening[All Fields] AND ("mothers"[MeSH Terms] OR "mothers"[All Fields] OR "maternal"[All Fields]) AND conditions[All Fields]) AND ("open access"[filter] AND ("2009/01/01"[PubDate] : "2023/06/01"[PubDate]))

Number of articles searched = 9403

3. POPLINE

Number of articles searched = 322

4. **Science Direct website**

Number of articles searched = 103

5. **Google Scholar**

Number of articles searched = 28
