# Supplementary material for: Global burden of potentially life-threatening maternal conditions: a systematic review and meta-analysis
Source: BMC Pregnancy Childbirth. 2024 Jan 2;24:11. doi: 10.1186/s12884-023-06199-9 (PMC10759711; doi:10.1186/s12884-023-06199-9)
Supplement: Supplementary file 4 — Additional file 4. [file 12884_2023_6199_MOESM4_ESM.pdf]

S4 Table: JBI Critical Appraisal Checklist for observational study

S4.1 Table: JBI Critical Appraisal Checklist for Cohort Studies

**JBI Critical Appraisal Checklist for Cohort Studies (Yes=Y, No=N, Unclear =UC, Not applicable=NA)**	**Alvaro J 2014**	**Crom A, 2016**	**Nam J, 2019**	**Magar J, 2020**	**Beyene T, 2022**	**Tura, A 2018**	**Nam J, 2022**
Were the two groups similar and recruited from the same population?	Y	Y	Y	Y	Y	Y	Y
Were the exposures measured similarly to assign people to both exposed and unexposed groups?	Y	Y	Y	Y	Y	Y	Y
Was the exposure measured in a valid and reliable way?	Y	Y	Y	Y	Y	Y	Y
Were confounding factors identified?	Y	Y	Y	Y	N	N	Y
Were strategies to deal with confounding factors stated?	Y	Y	Y	Y	N	N	Y
Were the groups/participants free of the outcome at the start of the study (or at the moment of exposure)?	Y	Y	Y	Y	Y	Y	Y
Were the outcomes measured in a valid and reliable way?	Y	Y	Y	Y	Y	Y	Y
Was the follow up time reported and sufficient to be long enough for outcomes to occur?	Y	Y	Y	Y	Y	Y	Y
Was follow up complete, and if not, were the reasons to loss to follow up described and explored?	N	Y	Y	Y	Y	Y	Y
Were strategies to address incomplete follow up utilized?	N	Y	Y	Y	Y	Y	Y
Was appropriate statistical analysis used?	Y	Y	Y	Y	Y	Y	Y
**Total Yes**	**9**	**11**	**11**	**11**	**9**	**9**	**11**

S4.2 Table: JBI Critical Appraisal Checklist for Analytical Cross Sectional Studies

JBI Critical Appraisal Checklist for Analytical Cross Sectional Studies (Yes=√, No=x, Unclear =uc, Not applicable=NA)	Chb M, 2015	Santana,	Moreira D,2017	Aleman A 2022	Owolabi O, 2020	Woldeyes W, 2018	Tenaw S, 2021	Herklots T, 2017	Hitti J, 2018	Rajbanshi S, 2021	Santana D, 2017	Norhayati M, 2016	Reid L, 2018	Norhayati M, 2016	Dzakpasu S, 2020	Tunçalp O, 2014
1. Were the criteria for inclusion in the sample clearly defined?	Y	Y	y	Y	y	y	y	y	y	y	y	y	y	y	y	y
2. Were the study subjects and the setting described in detail?	Y	Y	y	Y	y	y	y	y	y	y	y	y	y	y	y	y
3. Was the exposure measured in a valid and reliable way?	y	Y	y	y	y	y	y	y	y	y	y	y	y	y	y	y
4. Were objective, standard criteria used for measurement of the condition?	Y	Y	y	y	y	y	y	y	y	y	y	y	y	y	y	y
5. Were confounding factors identified?	N	N	y	n	n	y	y	n	n	y	y	n	y	y	n	y
6. Were strategies to deal with confounding factors stated?	N	N	y	n	n	y	y	n	n	y	y	n	y	y	n	y
7. Were the outcomes measured in a valid and reliable way?	Y	Y	y	y	y	y	y	y	y	y	y	y	y	y	y	y
8. Was appropriate statistical analysis used?	Y	Y	y	y	y	y	y	y	y	y	y	y	y	y	y	y
**Overall appraisal**	6	6	8	6	6	8	8	6	6	8	8	6	8	8	6	8

Continue

JBI Critical Appraisal Checklist for Analytical Cross Sectional Studies (Yes=√, No=x, Unclear =uc, Not applicable=NA)	Pacagnella , 2014	Oliveira F, 2014	Ghazivakili Z, 2016	Serruya S, 2017	Teka H ,2022	Roopa PS, 2013	Tunçalp O, 2013	Maity S, 2022	Balachandran D,2022	Ba, Anna C, 2021	Tan J, 2015	Jabir M2013	Tallapureddy S, 2017	Francisco A,2018	Murki A, 2017	Menezes et al. 2015
1. Were the criteria for inclusion in the sample clearly defined?	y	y	y	y	y	y	y	y	y	y	y	y	y	y	y	y
2. Were the study subjects and the setting described in detail?	y	y	y	y	y	y	y	y	y	y	y	y	y	y	y	y
3. Was the exposure measured in a valid and reliable way?	y	y	y	y	y	y	y	y	y	y	y	y	y	y	y	y
4. Were objective, standard criteria used for measurement of the condition?	y	y	y	y	y	y	y	y	y	y	y	y	y	y	y	y
5. Were confounding factors identified?	y	y	n	y	n	n	n	n	n	y	y	n	n	n	n	y
6. Were strategies to deal with confounding factors stated?	n	n	n	y	n	n	n	n	n	y	y	n	n	n	n	y
7. Were the outcomes measured in a valid and reliable way?	n	n	y	y	y	y	y	y	y	y	y	y	y	y	y	y
8. Was appropriate statistical analysis used?	y	y	y	y	y	y	y	y	n	y	y	y	y	y	y	y
**Overall appraisal**	6	6	6	8	6	6	6	6	6	8	8	6	6	6	6	8

S4.3 Table: JBI Critical Appraisal Checklist for Case Control Studies

**JBI Critical Appraisal Checklist for Case Control Studies (Yes=√, No=x, Unclear =uc, Not applicable=NA)**	**Paes L, 2014**	Madeiro A,2015	Raineau M, 2022	Chhabra P,2019	Fauconnier A, 2020
Were the groups comparable other than the presence of disease in cases or the absence of disease in controls?	y	y	y	y	y
Were cases and controls matched appropriately?	n	y	y	y	y
Were the same criteria used for identification of cases and controls?	y	y	y	y	y
Was exposure measured in a standard, valid and reliable way?	y	y	y	y	y
Was exposure measured in the same way for cases and controls?	y	y	y	y	y
Were confounding factors identified?	y	n	y	y	n
Were strategies to deal with confounding factors stated?	y	n	y	y	n
Were outcomes assessed in a standard, valid and reliable way for cases and controls?	y	y	y	y	y
Was the exposure period of interest long enough to be meaningful?	y	y	y	y	y
Was appropriate statistical analysis used?	y	y	y	y	y
**Total score out of 10**	**9**	**9**	**10**	**10**	**9**
