# Supplementary figures and images for: Global burden of potentially life-threatening maternal conditions: a systematic review and meta-analysis
Source: BMC Pregnancy Childbirth. 2024 Jan 2;24:11. doi: 10.1186/s12884-023-06199-9 (PMC10759711; doi:10.1186/s12884-023-06199-9)

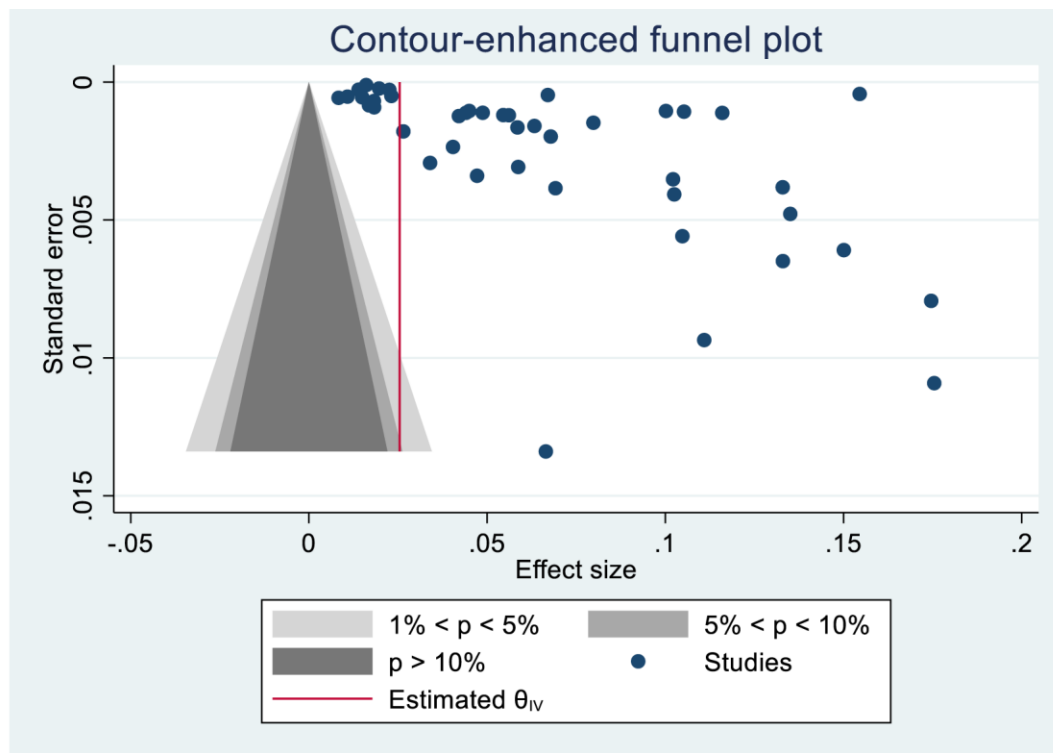

S5 Fig. Funnel plot of publication bias of the potentially life-threatening conditions

Supplement: Supplementary file 5 — Additional file 5. [file 12884_2023_6199_MOESM5_ESM.pdf]

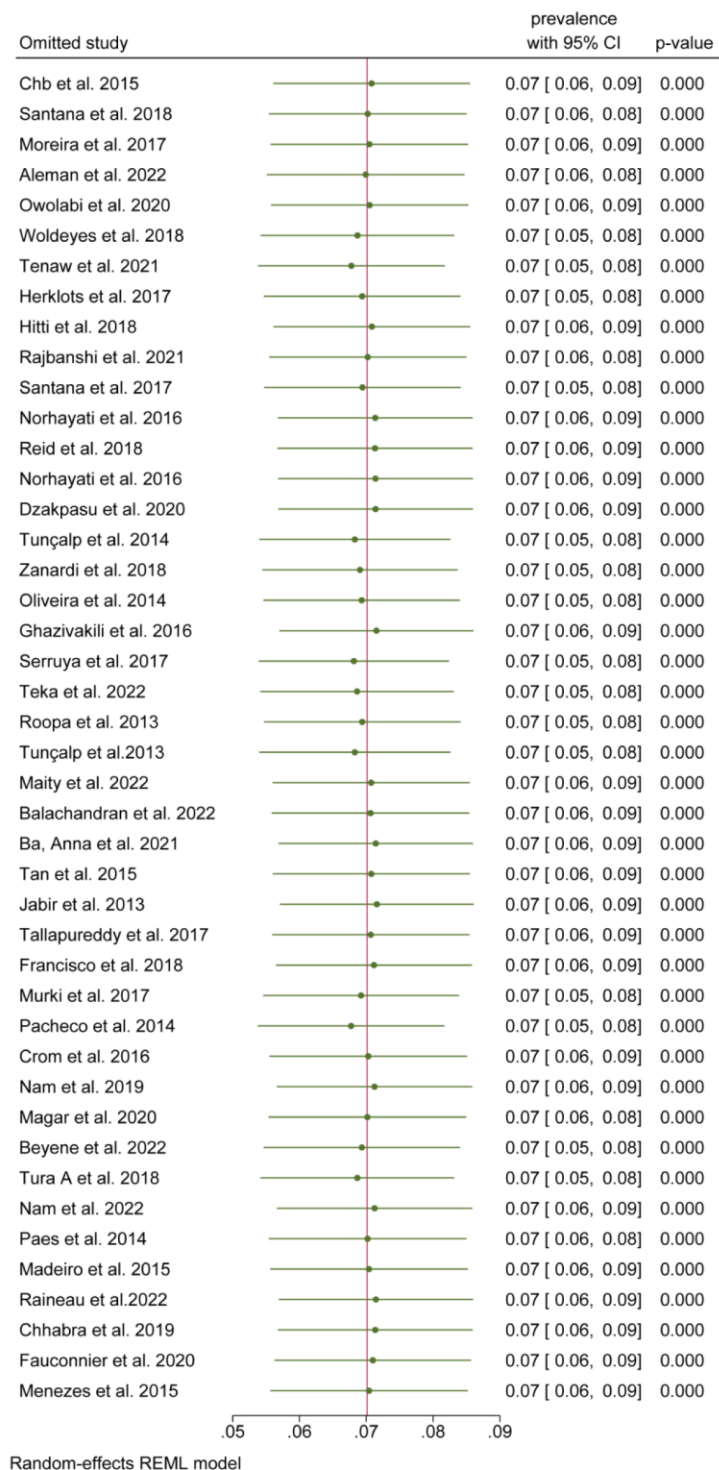

S6 Fig. Forest plot showing leave-one-out for prevalence of potentially life-threatening

Supplement: Supplementary file 6 — Additional file 6. [file 12884_2023_6199_MOESM6_ESM.pdf]
